# Dominant CD8^+^ T Cell Nucleocapsid Targeting in SARS-CoV-2 Infection and Broad Spike Targeting From Vaccination

**DOI:** 10.3389/fimmu.2022.835830

**Published:** 2022-02-22

**Authors:** Ellie Taus, Christian Hofmann, Francisco Javier Ibarrondo, Mary Ann Hausner, Jennifer A. Fulcher, Paul Krogstad, Kathie G. Ferbas, Nicole H. Tobin, Anne W. Rimoin, Grace M. Aldrovandi, Otto O. Yang

**Affiliations:** ^1^ Department of Molecular and Medical Pharmacology, David Geffen School of Medicine, University of California, Los Angeles, Los Angeles, CA, United States; ^2^ Department of Medicine, David Geffen School of Medicine, University of California Los Angeles, Los Angeles, CA, United States; ^3^ Department of Pediatrics, David Geffen School of Medicine, University of California Los Angeles, Los Angeles, CA, United States; ^4^ Fielding School of Public Health, University of California Los Angeles, Los Angeles, CA, United States; ^5^ Department of Microbiology, Immunology, and Molecular Genetics, David Geffen School of Medicine, University of California Los Angeles, Los Angeles, CA, United States

**Keywords:** SARS-CoV-2, cellular immunity, CD8+ T cells, COVID-19 vaccine, COVID-19

## Abstract

CD8^+^ T cells have key protective roles in many viral infections. While an overall Th1-biased cellular immune response against SARS-CoV-2 has been demonstrated, most reports of anti-SARS-CoV-2 cellular immunity have evaluated bulk T cells using pools of predicted epitopes, without clear delineation of the CD8^+^ subset and its magnitude and targeting. In recently infected persons (mean 29.8 days after COVID-19 symptom onset), we confirm a Th1 bias (and a novel IL-4-producing population of unclear significance) by flow cytometry, which does not correlate to antibody responses against the receptor binding domain. Evaluating isolated CD8^+^ T cells in more detail by IFN-γ ELISpot assays, responses against spike, nucleocapsid, matrix, and envelope proteins average 396, 901, 296, and 0 spot-forming cells (SFC) per million, targeting 1.4, 1.5, 0.59, and 0.0 epitope regions respectively. Nucleocapsid targeting is dominant in terms of magnitude, breadth, and density of targeting. The magnitude of responses drops rapidly post-infection; nucleocapsid targeting is most sustained, and vaccination selectively boosts spike targeting. In SARS-CoV-2-naïve persons, evaluation of the anti-spike CD8^+^ T cell response soon after vaccination (mean 11.3 days) yields anti-spike CD8^+^ T cell responses averaging 2,463 SFC/million against 4.2 epitope regions, and targeting mirrors that seen in infected persons. These findings provide greater clarity on CD8^+^ T cell anti-SARS-CoV-2 targeting, breadth, and persistence, suggesting that nucleocapsid inclusion in vaccines could broaden coverage and durability.

## Introduction

The correlates of immune protection against SARS-CoV-2 are still being defined (1, 2). Antibodies likely prevent or lessen early infection (3–5), but have little capacity to ameliorate established severe infection (6–8). The mRNA vaccines afford protection from disease after a single dose before detectable neutralizing antibodies, indicating importance of cellular immunity (2, 9). Likely both antibodies and T cells have important roles, separately or in concert.

A global Th1 T cell profile correlates with positive outcome after infection (10), including bias of virus-specific T cells (11–14). Development of virus-specific cellular immunity correlates to recovery from infection (12, 15). Given the protective role of CD8^+^ T cells in many viral infections, SARS-CoV-2-specific CD8^+^ T cells (16–19) may be particularly important for preventing severe disease. Antiviral CD8^+^ T cell frequency and breadth have not been clearly defined in most studies, most of which have used single pools of predicted epitopes from across the proteome and/or unseparated PBMC for qualitative evaluations.

SARS-CoV-2 vaccines protect against serious illness and death (20, 21). There have been increasing observations of vaccinated persons becoming infected, associated with viral spike mutations mediating antibody resistance (22–25). Despite these breakthrough infections, vaccination still protects against severe illness or death, further underscoring the importance of cellular immunity (9, 26). To date, however, CD8^+^ T cell responses against the vaccine have not been compared in detail to natural infection.

Here we confirm the overall Th1 profile of SARS-CoV-2-specific T cells and examine the targeting of spike, nucleocapsid, matrix, and envelope proteins by the CD8^+^ T cell subset in persons recovered from recent SARS-CoV-2 infection. The stability of these responses is evaluated, as well as boosting after vaccination. In SARS-CoV-2-naïve individuals, vaccine-elicited antiviral CD8^+^ T cell targeting is compared to that from natural infection.

## Materials And Methods

### Study Participants and Samples

Participants with known immunocompromising conditions (including diabetes mellitus, immunosuppressive medications, HIV-1 infection) were excluded. All COVID-19 recovered persons were infected no later than January 2021, and the majority had mild infection (not requiring supplemental oxygenation or hospitalization). PBMC were isolated by density gradient centrifugation and viably cryopreserved until use.

### SARS-CoV-2 Synthetic Peptides for Intracellular Cytokine Staining Assays

For intracellular cytokine staining assays, synthetic peptide “megapools” (27) were generously provided by D. Weiskopf and A. Sette. The sets of predicted CD4^+^ T cell epitopes and overlapping spike peptides were combined in one pool, and the two sets of predicted CD8^+^ T cell epitopes were combined in a second pool. The final concentration of each peptide during PBMC stimulation was 1µg/ml.

### Intracellular Cytokine Staining (ICS) Flow Cytometry

ICS was performed as described (28) with modifications. Cryopreserved PBMC were thawed and plated at ~5x10^5^ cells per well in 96 well U-bottom plates, with brefeldin A (#00-4506-51, eBioscience, San Diego, CA) and monensin (#00-4505-51, eBioscience, San Diego, CA) per manufacturer’s directions. Each PBMC sample had four wells with: spike plus CD4^+^ T cell epitope megapools, CD8^+^ T cell epitope megapools, no additive, or 1µg/ml ionomycin and 500ng/ml PMA (#407951 and #524400, Calbiochem, San Diego, CA). After 6 hours at 37°C, cells were transferred to 5ml polystyrene tubes, washed in PBS with 2% heat inactivated fetal calf serum (wash buffer), and resuspended in wash buffer including antibodies against CD3, CD8, CD4, and Fixable Aqua viability dye for 30 minutes at 4°C (**Supplementary Table S1**). After washing, cells were permeabilized with Foxp3/Transcription Factor Staining Buffer (#00-5523-00, eBioscience, San Diego, CA) according to manufacturer’s instructions, then stained with antibodies against IL-4, IL-2, IL-10, IFN-γ, and IL-17 (**Supplementary Table S1**) at room temperature for 30 minutes. After washing, the cells were fixed in PBS with 1% paraformaldehyde for analysis (**Supplementary Figure S1**) on an Attune NxT flow cytometer (ThermoFisher Scientific, West Hills, CA). A minimum of 54,000 live cell events were analyzed. Analysis was performed using FlowJo version 10 software (BD Biosciences).

### Determination of Serum Anti-RBD IgG Levels

Anti-RBD IgG levels were assessed as described (29). Briefly, 96-well microtiter plates were coated with 2 μg/mL recombinant RBD protein and blocked with 3% dried milk (Bioworld, Dublin, OH). Serum was added in duplicate serial dilutions, and bound antibodies were detected using goat anti-human IgG conjugated with horseradish peroxidase (Bethyl Laboratories, Montgomery, TX), followed by tetramethylbenzidine substrate solution (ThermoFisher Scientific, Waltham, MA) for measurements at 450 and 650 nm (Spark 10M, Tecan, Baldwin Park, CA). Each plate contained a control titration of the anti-RBD monoclonal antibody CR3022 (Creative Biolabs, Shirley, NY) to provide a standard curve. Serum anti-RBD IgG binding activity was expressed as an equivalent to a concentration of CR3022.

### SARS-CoV-2 Synthetic Peptides for IFN-γ ELISpot Assays

Synthetic overlapping peptides spanning SARS-CoV-2 spike, nucleocapsid, matrix, and envelope were obtained from BEI Resources (NR-52402, NR-52404, NR-52403, NR-52405). Lyophilized peptides were initially suspended in DMSO at 20mg/ml, then diluted 10x with water to 2mg/ml. Peptide pools were generated as in **Supplementary Table S2** at 100µg/ml each peptide, and the final concentration of each peptide during the ELISpot assay was 5µg/ml.

### IFN-γ ELISpot Assays for CD8^+^ T Cell Responses

These assays were performed as previously described for measuring HIV-1-specific responses using polyclonally expanded CD8^+^ T cells (30–33), which we and others have shown to correlate well to unexpanded fresh CD8^+^ T cells (30, 34). In brief, thawed cryopreserved PBMC were plated at 1 to 2 million cells/well in RPMI with IL-2 at 50U/ml (NIH AIDS Reagent Repository Program) with a CD3:CD4 bi-specific monoclonal antibody (gift of Dr. J Wong) and cultured for approximately 14 days to yield purified polyclonal CD8^+^ T cells. These cells were viably cryopreserved until the day of ELISpot assay. Cells were added to a 96-well filter plate that had been pre-coated with an anti-IFN-γ antibody (#3420-3-1000, Mabtech, Nacka Strand, Sweden) with the addition of a peptide pool, medium alone (three wells), or medium with PHA (#L1668, Sigma Aldrich, St. Louis, MO) at 25µg/ml. After overnight incubation in a humidified CO_2_ incubator, the plate was washed and stained with biotinylated anti-IFN-γ antibody (#3420-6-250, Mabtech, Nacka Strand, Sweden) for visualization using a streptavidin-peroxidase reagent and counting on an automated ELISpot reader (AID, Autoimmun Diagnostika GMBH, Strassberg, Germany). The response against each peptide pool was expressed as the raw count minus the mean of the triplicate negative control wells. A positive response against a peptide pool was defined as both ≥50 SFC/million cells and ≥ the mean of the negative control wells plus three standard deviations.

### Statistics

Statistical comparisons (two-tailed heteroscedastic Student’s t-test) and graphs utilized Microsoft Excel for Mac version 16.50. Sørenson similarity indices between ELISpot assays were calculated as: the total number of shared spike pool responses (defined as above) ÷ the total number of spike peptide pools (twelve).

### Study Approval

Prior to participation, all participants gave written informed consent under an institutional review board-approved protocol at the University of California Los Angeles.

## Results

### After Infection, There Are Predominately IFN-γ-Expressing CD4^+^ T Cell Responses Against SARS-CoV-2

**Figure 1:**
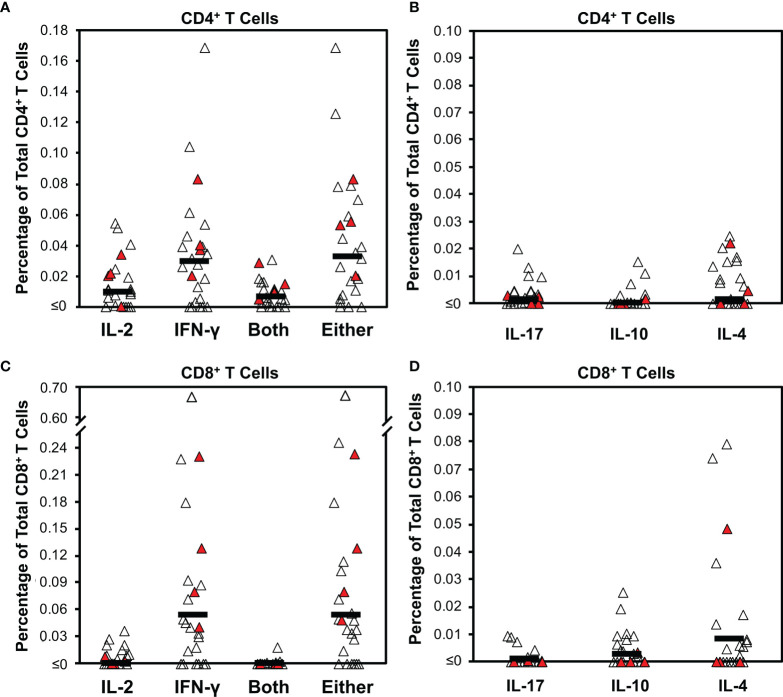
Intracellular cytokine staining for T cell responses against SARS-CoV-2 early after infection demonstrates bias for IFN- γ production. Cytokine production was determined by intracellular cytokine staining for CD4^+^ and CD8^+^ T cell subsets after stimulation with a pool of overlapping peptides spanning spike combined with predicted CD4^+^ epitopes from across the proteome (**Supplementary Figure S1**) for 25 persons (21 with mild infection, 4 with severe infection) a mean of 29.8 days from COVID-19 symptom onset (range 15 to 49 days). Filled symbols indicate persons who had severe infection. **(A)** The background-subtracted frequencies of CD4^+^ T cells producing IL-2, IFN-γ, both cytokines, or either cytokine are plotted. Dark horizontal bars indicate means, which were 0.010%, 0.030%, 0.007%, and 0.033%, respectively. Defining responses as being ≥0.01% above background, responders for these four cytokine response groupings were 12/25 (48%), 17/25 (68%), 10/25 (40%), and 18/25 (72%), respectively. **(B)** The background-subtracted frequencies of CD4^+^ T cells producing IL-17, IL-10, or IL-4 are plotted. Dark horizontal bars indicate means, which were 0.003%, 0.002%, and 0.007%, respectively. Defining responses as being ≥0.01% above background, responders for these three cytokine responses were 3/26 (11.5%), 2/26 (7.7%), 10/25 (40%), and 7/26 (26.9%), respectively. **(C)** The background-subtracted frequencies of CD8^+^ T cells producing IL-2, IFN-γ, both cytokines, or either cytokine are plotted. Dark horizontal bars indicate means, which were 0.001%, 0.053%, 0.000%, and 0.054%, respectively. Defining responses as being ≥0.01% above background, responders for these four cytokine response groupings were 6/25 (24%),17/25 (68%), 1/25 (4%), and 20/25 (80%), respectively. **(D)** The background-subtracted frequencies of CD8^+^ T cells producing IL-17, IL-10, or IL-4 are plotted. Dark horizontal bars indicate means, which were 0.002%, 0.004%, and 0.012% respectively. Defining responses as being ≥0.01% above background, responders for these three cytokine responses were 0/26 (0%), 3/26 (11.5%), 10/25 (40%), and 6/26 (23.1%), respectively.

**Figure 2:**
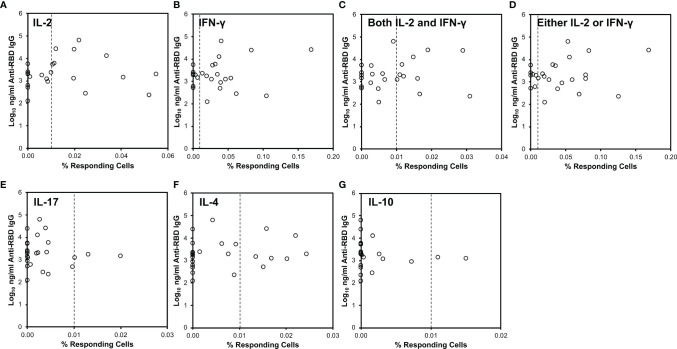
CD4^+^ T cell cytokine responses against SARS-CoV-2 do not correlate to serum anti-RBD antibody levels. SARS-CoV-2-specific responses defined as in **Figure 1** (x-axis) were compared to serum anti-RBD IgG antibody levels (y-axis). The vertical dotted line indicates 0.01% responding cells producing the indicated cytokine(s). **(A)** Relationship to IL-2-producing cells. **(B)** Relationship to IFN-γ-producing cells. **(C)** Relationship to cells producing both IL-2 and IFN-γ. **(D)** Relationship to cells producing either IL-2 or IFN-γ or both. **(E)** Relationship to IL-17-producing cells. **(F)** Relationship to IL-4-producing cells. **(G)** Relationship to IL-10-producing cells.

Virus-specific T cell responses in 25 persons early after COVID-19 (mean 29.8 days after symptom onset, range 15-49 days) were assessed by intracellular cytokine staining after stimulating PBMC with pooled peptides. These peptides spanned the spike protein and included predicted CD4^+^ T cell epitopes across the proteome (spike and CD4 “megapool peptides” (27). CD4^+^ T cells were assessed for IL-2, IFN-γ, IL-4, IL-10, or IL-17 production (**Supplementary Figure S1** and **Figures 1A, B**). IFN-γ production predominated, with a mean of 0.030% positive cells (17/25, 68% of persons above 0.01%). IL-2 responses were lower, with a mean of 0.010% (12/25, 48% of persons above 0.01%). Most cells producing IL-2 also produced IFN-γ; the mean percentage of cells producing either was 0.033% (18/25, 72% of persons above 0.01%). Few virus-specific CD4^+^ T cells produced IL-10, IL-17, or IL-4 (means 0.003%, 0.002%, and 0.007%, respectively). None of these responses correlated to concurrent anti-RBD antibody levels (**Figure 2**).

### CD8^+^ T Cell Responses Against SARS-CoV-2 Are Also Predominately IFN-γ-Expressing

The CD8^+^ T cell subset was evaluated in parallel (**Supplementary Figure S1** and **Figures 1C, D**). The response was mostly limited to IFN-γ, with a mean of 0.053% positive cells (17/25 or 68% above 0.01%). IL-2 responses were minimal, with a mean of 0.001% (6/25 or 18% of persons above 0.01%). Again, IL-2 production mostly overlapped IFN-γ production; the mean percentage of cells producing either was 0.054% positive cells (18/25, 72% of persons above 0.01%). There were minimal IL-17, IL-10, or IL-4 responses (means 0.002%, 0.004%, and 0.012%, respectively), but a few individuals had significant IL-4 responses (6/26, 23% of persons above 0.01%). None of these responses correlated to anti-RBD antibody levels (**Supplementary Figure S2**). Testing of intracellular cytokine responses using pooled peptides of predicted CD8^+^ minimal epitopes from across the SARS-CoV-2 proteome (CD8 “megapools” (27) yielded lower frequencies with a similar pattern (**Supplementary Figure S3**).

### CD8^+^ T Cell Responses Broadly Target Nucleocapsid, Spike, and Matrix, but Not Envelope, and Nucleocapsid Is Immunodominant

**Figure 3:**
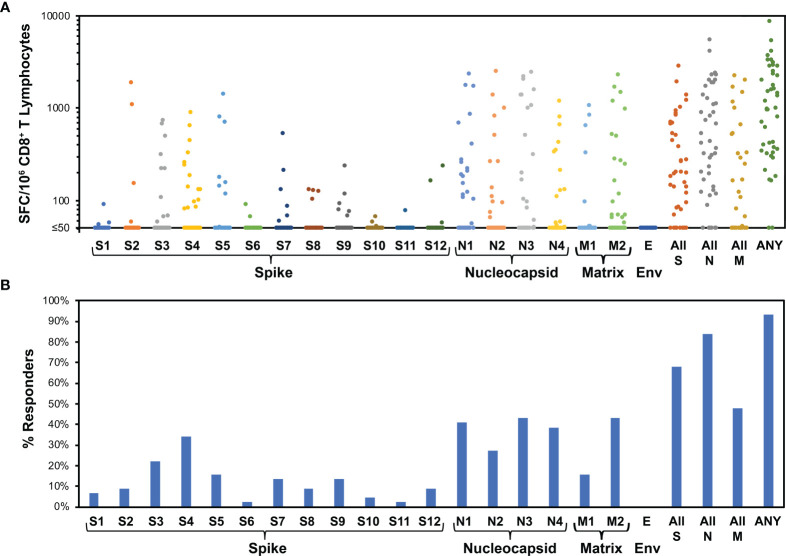
Evaluation of CD8^+^ T cell targeting of SARS-CoV-2 by ELISpot using peptide pools demonstrates broad targeting of spike, nucleocapsid, and matrix, with dominance of nucleocapsid targeting. For 44 persons after recent SARS-CoV-2 infection (36 with mild infection, 8 with severe infection, mean 31.1 days, range 11 to 47 days after symptom onset), IFN-γ ELISpot was performed on polyclonally expanded CD8^+^ T cells using peptides spanning spike, nucleocapsid, matrix, and envelope proteins, which were combined in pools of 16 or fewer (**Supplementary Table 1**). Spike was contained in 12 pools (S1 to S12), nucleocapsid in four pools (N1 to N4), matrix in two pools (M1 to M2), and envelope in one pool (E). **(A)** Frequencies of responses against each pool are plotted for each participant. The mean total responses against spike, nucleocapsid, matrix, and envelope were 396 SFC/million CD8^+^ T cells, 901 SFC/million CD8^+^ T cells, 296 SFC/million CD8^+^ T cells, and 0 SFC/million CD8^+^ T cells, respectively. **(B)** Percentages of persons responding against each pool are plotted. Targeting of spike, nucleocapsid, matrix, and envelope was an average of 1.4, 1.5, 0.6, and 0.0 peptide pools per person, respectively. Response against pools S4 and S5, comprising the receptor binding domain of spike, was an average 0.5 peptide pools per person.

The CD8^+^ T cell responses were studied at higher resolution using IFN-γ ELISpot assays for responses to smaller pools of overlapping peptides spanning spike (12 pools), nucleocapsid (four pools), matrix (two pools), and envelope (one pool) proteins. This assay yielded spike-specific responses correlating to intracellular cytokine staining IFN-γ responses to stimulation with the spike/CD4^+^ T cell megapool (**Supplementary Figure S4**). Across individuals, all pools were targeted except envelope (**Figure 3**). The average total responses against spike, nucleocapsid, matrix, and envelope were 396, 901, 296, and 0 spot-forming cells (SFC) per million CD8^+^ T cells, respectively. Targeting density considered in relationship to target protein size yielded means of 0.31, 2.15, and 1.33 SFC/million CD8^+^ T cells/amino acid against spike, nucleocapsid, and matrix, respectively (**Supplementary Figure S5A**). Targeting of nucleocapsid was significantly greater than spike (p<0.0001) but not significantly greater than matrix (p=0.15), while matrix targeting was also significantly greater than spike (p=0.012). A similar hierarchy was noted for numbers of responses against pools, a surrogate for breadth of epitope targeting. On average, each person targeted 1.43, 1.50, 0.59, and 0 pools in spike, nucleocapsid, matrix, and envelope, respectively. Assuming that each recognized pool corresponded to one recognized epitope, this equated to 0.0011, 0.0036, and 0.0027 epitopes targeted per amino acid for spike, nucleocapsid, and matrix, respectively (**Supplementary Figure S5B**). Epitope targeting of nucleocapsid was significantly greater than spike (p<0.0001) but not significantly greater than matrix (p=0.11), while matrix targeting was also significantly greater than spike (p=0.0029). Finally, comparisons of CD8^+^ T cell targeting to anti-RBD antibody levels revealed no correlation (**Supplementary Figure S6**). Overall, these findings demonstrated highly dominant CD8^+^ T cell targeting of nucleocapsid over spike, likely intermediate targeting of matrix, and no targeting of envelope.

### CD8^+^ T Cell Responses Against Spike, Nucleocapsid, and Matrix Generally Wane Over Time, and Responses Against Nucleocapsid Are More Persistent

**Figure 4:**
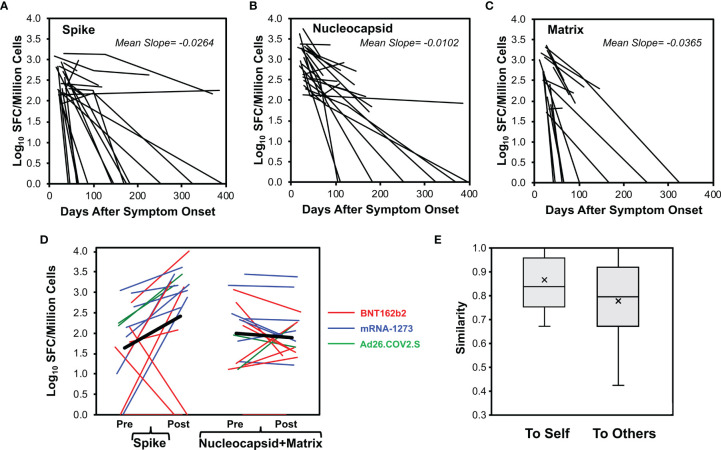
CD8^+^ T cell responses decay after SARS-CoV-2 infection but vaccination boosts memory against spike protein. CD8^+^ T cell responses were measured longitudinally by ELISpot assay in 29 persons monitored starting early SARS-CoV-2 infection (23 with mild infection, 6 with severe infection, starting <45 days after symptom onset), serial measurements are plotted for 23 total spike responses **(A)**, 24 total nucleocapsid responses **(B)**, and 16 total matrix responses **(C)**. **(D)** For 17 persons with prior COVID-19 who were vaccinated with an available pre-vaccination measurement within 65 days (14 with mild infection, 3 with severe infection including one who had critical infection, vaccinated mean of 225 days post onset of symptoms, range 64 to 394 days), baseline pre-vaccination (mean of -21.9 days, range -63 to +3 days before vaccination) and resulting post-vaccination (first dose, mean of 12.8 days, range 5 to 29 days after vaccination) total response levels against spike and combined nucleocapsid plus matrix are plotted. Eight vaccinees received BNT162b2 (red), seven vaccinees received mRNA-1273 (blue), and two vaccinees received Ad26.COV2.S (green). Two non-responders had no detectable response at baseline and received BNT162b2. One non-responder had prior severe illness and the remainder had mild illness. **(E)** For the vaccinated persons, Sørenson similarity values were calculated between pre- and post- vaccination recognized spike pools within each person (self) and across all combinations with other persons (others). Box plots indicate 25th to 75th quartiles and medians, with medians (horizontal line) and means (x) marked. The high background similarity between individuals resulted from the high number of unrecognized pools (average 10.2/12 pools) and thus multiple shared unrecognized pools across persons.

For 29 persons with longitudinal measurements after early infection, responses were tracked for stability. There were 23 (**Figure 4A**), 24 (**Figure 4B**), and 16 (**Figure 4C**) responders available to evaluate for spike, nucleocapsid, and matrix responses, respectively. These responses generally waned over time, with drops in 21/23 (91%), 23/24 (96%), and 15/16 (94%), respectively. Because they often fell to undetectable levels by the second measurement, calculated decay rates were minimal estimates; the observed mean slopes were -0.026, -0.010, and -0.037 log_10_ SFC/million CD8^+^ T cells/day for spike, nucleocapsid, and matrix, respectively. Comparing these slopes, loss of anti-nucleocapsid responses was slower than anti-spike (p=0.042) and anti-matrix (p=0.018) responses. Thus, CD8^+^ T cell responses against nucleocapsid were not only immunodominant, but more persistent.

### SARS-CoV-2 Vaccines Boost Memory CD8^+^ T Cell Responses Against Spike

The mRNA and adenovirus-based COVID-19 vaccines would be expected to access the human leukocyte antigen class I pathway to elicit CD8^+^ T cell responses. Anti-spike responses in 17 persons with past SARS-CoV-2 infection were evaluated pre- and post- vaccination. Vaccination occurred a mean of 225 days from symptom onset (range 64 to 394 days), with eight persons receiving BNT162b2, seven persons receiving mRNA-1273, and two persons receiving Ad26.COV2.S vaccines. In 13/17 persons (76%), the magnitude of anti-spike responses increased after vaccination (**Figure 4D**). Responses against nucleocapsid and matrix fell in 12/17 persons (71%). Increases in responses against nucleocapsid and matrix tended to be observed at the low end of assay sensitivity (~100 SFC/million CD8^+^ T cells), suggesting assay noise. Among the four persons in whom spike responses did not increase after vaccination, two had no detectable responses at baseline before vaccination, and all four received BNT162b2. Comparison of peptide pool targeting pre- and post- vaccination (**Figure 4E**) demonstrated significantly greater Sørenson similarity indices within individuals than between individuals (means 0.86 and 0.78, respectively, p=0.010). These findings confirmed that vaccination yields spike targeting similar to prior infection, indicative of boosting memory responses.

### SARS-CoV-2 Vaccines Elicit Spike-Specific CD8^+^ T Cell Responses With Similar Targeting in Previously Uninfected Persons Compared to Natural Infection

**Figure 5:**
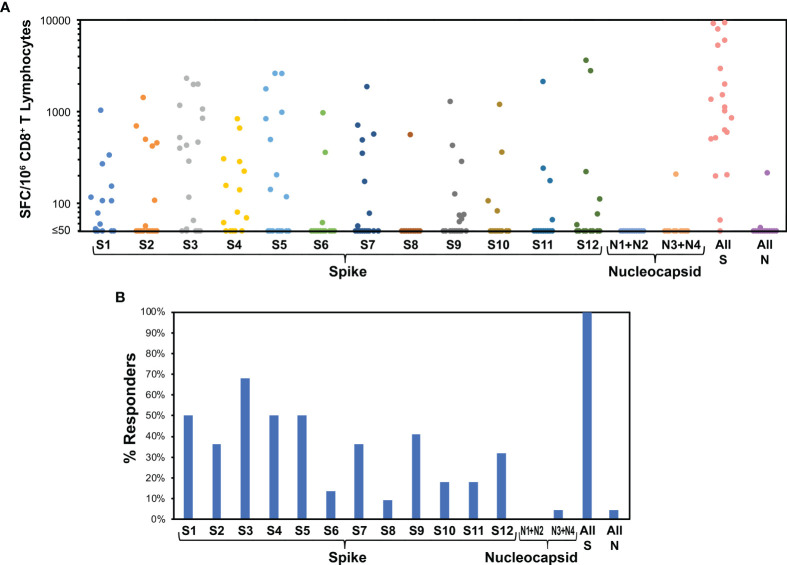
Spike targeting after vaccination of persons without prior SARS-CoV-2 infection is broadly distributed. 22 persons without a history of SARS-CoV-2 infection were monitored for responses against spike and nucleocapsid (negative control) by ELISpot assay after vaccination with BNT162b2 (15 persons) or mRNA-1273 (7 persons). Responses were evaluated a mean of 11.3 days after the first vaccine dose (range 8 to 16 days). **(A)** Frequencies of responses against each pool are plotted for each participant. The mean total response against spike was 2,463 SFC/million CD8^+^ T cells. **(B)** Percentages of persons responding against each pool are plotted. Targeting of spike was an average of 4.2 pools per person. Response against pools S4 and S5, comprising the receptor binding domain of spike, was an average 1.0 peptide pools per person.

**Figure 6:**
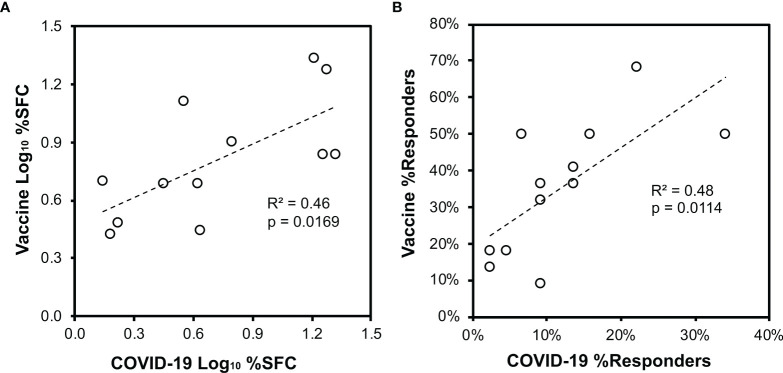
Vaccination of persons without prior SARS-CoV-2 infection elicits CD8^+^ T cell targeting of spike similar to natural infection. Across the 44 persons with recent SARS-CoV-2 infection (**Figure 3**) and 22 persons after vaccination without prior SARS-CoV-2 infection (**Figure 5**), CD8^+^ T cell responses against spike defined by ELISpot were compared. Pearson correlation p values are indicated. **(A)** The mean percentage contribution of each pool to the total spike response (log_10_ transformed) is plotted between the two groups. **(B)** The percentage of persons responding against each pool is plotted between the two groups.

IFN-γ ELISpot assays for CD8^+^ T cell responses against spike were performed for 22 persons without prior SARS-CoV-2 infection who were vaccinated (15 with BNT162b2 and 7 with mRNA-1273 vaccines). Responses a mean of 11.3 days after the first vaccination (range 8 to 16 days) showed targeting against all peptide pools (**Figure 5**), and the average total spike targeting was 2,463 SFC/million CD8^+^ T cells. Each person recognized a mean of 4.2 spike pools (range 1 to 10). Comparison to natural infection (**Figure 3**) in terms of the distribution of targeting (mean percentage of SFC against each pool versus entire spike, **Figure 6A**) or the frequency of targeting (percentage of persons recognizing each pool, **Figure 6B**) showed direct correlation, indicating that targeting induced by vaccination is similar to natural infection.

## Discussion

The protective contribution of SARS-CoV-2-specific CD8^+^ T cells is increasingly apparent (16–19), consistent with their known importance for clearing infected cells in other viral infections. Most studies have focused on the phenotypic characteristics of cellular immunity in bulk, such as cytokine production in response to pooled predicted epitope peptides, without detail on targeting of responses. We confirm Th1 bias in both CD4^+^ and CD8^+^ virus-specific T cells (11–14), using intracellular staining for IFN-γ, IL-2, IL-4, IL-17, and IL-10.

For both T cell subsets IFN-γ production dominated, followed by IL-2, with more CD4^+^ T cells than CD8^+^ T cells producing IL-2. Most IFN-γ-producing cells did not produce IL-2, particularly the CD8^+^ subset, supporting prior findings (35–37). Minimal production of IL-10 or IL-17 were observed; IL-10 (38) and IL-17 (39) production have been linked to disease progression, but our participants were mostly limited to those who recovered from mild illness.

A few individuals had significant populations of IL-4-producing CD8^+^ T cells of unclear significance, not previously reported in SARS-CoV-2 infection to our knowledge. IL-4-producing CD8^+^ T cells have been suggested to be noncytolytic helper cells that do not produce IFN-γ (40), associated with humoral immunity in old age (41), asthma in children (42), and autoimmune arthritis (43). Whether they might play a protective (anti-inflammatory), pathogenic (immunosuppressive), or mixed role in COVID-19 is unclear.

Our observations agree observations that most infected persons develop SARS-CoV-2-targeted cellular immune responses (36, 44–47). By intracellular cytokine staining flow cytometry with spike/CD4^+^ epitope megapools (27), 72% and 80% of persons had IFN-γ and/or IL-2 responses in the CD4^+^ and CD8^+^ T cell subsets, respectively. Considering all tested cytokines and both tested peptide megapools, all persons had both CD4^+^ and CD8^+^ T cell responses (not shown). By IFN-γ ELISpot for spike, nucleocapsid, matrix, and envelope, 93% of persons had detectable CD8^+^ T cell responses against at least one protein.

Others have observed preferential targeting of structural proteins (12, 14, 27, 35, 36, 44, 45, 48–50), using either predicted epitopes (12, 27, 45, 48, 50) or overlapping peptides (14, 35, 36, 44, 49) in pools. Two studies from Le Bert et al. (14) and Peng et al. (36) used multiple smaller pools in IFN-γ ELISpot assays to assess targeting breadth, but did not separate CD4^+^ from CD8^+^ T cell populations. Le Bert et al. found that responses against matrix were most targeted, followed by those against spike or nucleocapsid (envelope was not tested) (14), but Peng et al. found that spike was most highly targeted, while nucleocapsid and matrix were similar, and many persons had envelope targeting (36). However, the contributions of CD4^+^ versus CD8^+^ T cells to these patterns were not defined.

We follow up in greater detail using isolated CD8^+^ T cells with small pools of overlapping peptides spanning spike, nucleocapsid, matrix, and envelope in IFN-γ ELISpot assays of infected participants. In contrast to Le Bert et al. and Peng et al. using unseparated PBMC, we clearly identify nucleocapsid as the dominant target of CD8^+^ T cells. Matrix targeting is quantitatively similar to spike, but more densely targeted, and we observe no targeting of envelope. Similar to Le Bert et al. using unseparated PBMC, we find that targeting distributed across spike, and most persons target at least one spike epitope. Moreover, we observe modestly higher frequency of receptor binding domain (RBD) targeting, accounting for about a third of responses despite being about a sixth of spike. Even so, the few mutations mostly in the RBD defining various spike variants seem unlikely to affect recognition by CD8^+^ T cells (51). Overall, differences from the findings of Le Bert et al. and Peng et al. may result primarily from evaluation of isolated CD8^+^ T cells *versus* bulk PBMC (in which CD4^+^ T cells typically predominate); other studies grossly comparing CD4^+^ T cells to CD8^+^ T cells have shown that the former tend to predominate (12, 46, 49, 52–54), and that the two do not correlate (55). Finally, our results suggest that nucleocapsid might be a useful target for vaccine inclusion to elicit broader and more durable CD8^+^ T cell responses, which is also supported by a recent study suggesting that immunodominant targeting of nucleocapsid is associated with better outcome after SARS-CoV-2 infection (56).

Various studies reported differing results on longevity of virus-specific T cell responses (CD4^+^, CD8^+^ or combined) after SARS-CoV-2 infection, with observations of both persistence (37, 45, 47, 53–55, 57, 58) and decay (14, 47, 59, 60). We observed decay of CD8^+^ T cell responses similar to our observations of anti-RBD antibodies (29, 61), and most rapid waning of matrix responses, in agreement with Le Bert et al. (14). The reasons for discrepant findings between studies are unclear, but may relate to methodologies (intracellular cytokine staining versus ELISpot), CD4^+^ versus CD8^+^ versus unseparated subsets, targeting of responses measured, and differences in illness severity. As opposed to antibodies (and B cell memory) being required for immediate viral neutralization during exposure or early infection, T cells probably have a more prolonged effector role in containing and clearing infection, so it is unclear whether our observed peripheral blood decay of the CD8^+^ T cell response is functionally relevant. Data showing ongoing vaccine protection from severe illness or death despite waning protection from infection (26, 62) are further evidence that the falling level of circulating antiviral CD8^+^ T cells we observe do not preclude an effective recall response.

Some groups have observed either correlation (36, 63) or lack of correlation (47) of T cell responses to antibodies. We saw no correlation of either CD4^+^ or CD8^+^ T cell responses by intracellular cytokine staining, or CD8^+^ T cell responses by IFN-γ ELISpot to anti-RBD antibodies. These discrepancies again may be related to methodologies or clinical characteristics of the participants. Of note, our cohort included mostly persons who had mild infection (not requiring supplemental oxygen or hospitalization). Because antibody levels vary greatly by disease severity (29, 64–66), our dynamic range might have been too limited to see a correlation.

Our data address spike targeting induced by vaccination. While initial pilot studies of the two mRNA vaccines demonstrated cellular immunity (67, 68), these were measurements of whole PBMC by intracellular cytokine staining using a single peptide pool. We provide greater detail, showing vaccination elicits an average of over four targeted epitopes with summed frequency over 1,000 per million CD8^+^ T cells. While this breadth and frequency was higher than we observed for natural infection, our measurement after vaccination was at the peak, and infected persons were assessed past the peak during infection. A prior study provided results consistent with our suggestion that vaccination boosts prior memory responses against spike; in persons receiving the BNT162b2 vaccine, those with prior infection reached a level of spike targeting after a single dose that was attained after two doses in persons without prior infection (69). Qualitatively, however, we observed that vaccination and infection generated similar spike responses.

Our study has caveats. Evaluation of cytokine production and T cell “polyfunctionality” was limited to few cytokines and performed with too few PBMC for accurate quantitation or sensitivity below ~0.01%. Our evaluation of CD8^+^ T cells utilized a cell-sparing expansion method, although results with expanded cells have been shown to correlate well to bulk unexpanded CD8^+^ T cells (30, 34). Responses were evaluated only against spike, nucleocapsid, matrix, and envelope, and could miss dominant responses against other proteins. We did not map to the level of individual peptides/epitopes; thus, we likely underestimated the breadth and depth of targeting. The time points after infection were not frequent enough for precise estimation of decay rates, but only provide minimal boundaries for decay. Most of the COVID-19 participants had had mild illness, and there were too few severely ill subjects for comparisons. Finally, the numbers of participants were too small to compare responses between different vaccines.

In summary, we find a Th1-biased IFN-γ dominant cellular immune response after SARS-CoV-2 infection in both CD4^+^ and CD8^+^ subsets, although some persons have an unusual IL-4-producing CD8^+^ T cell population of unclear significance. CD8^+^ T cells predominately target nucleocapsid and those responses appear to be more durable compared to targeting of spike or matrix; no responses were seen against envelope. Vaccination of previously infected persons specifically boosts memory responses against spike, and generates new responses in previously uninfected persons that resemble those from infection. These results provide greater clarity on CD8^+^ T cell targeting, breadth, and persistence. Inclusion of nucleocapsid in vaccines may allow even broader and longer-lived cellular immune protection against COVID-19 to combat ongoing viral evolution in the pandemic.

## Data Availability Statement

The original contributions presented in the study are included in the article/**Supplementary Material**, further inquiries can be directed to the corresponding author.

## Ethics Statement

The studies involving human participants were reviewed and approved by UCLA Institutional Review Board. The patients/participants provided their written informed consent to participate in this study.

## Author Contributions

Overall study conceptualization: OY. Study design: ET, CH, FI, JF, PK, KF, NT, AR, GA, and OY. Conducting experiments: ET, CH, FI, and MH. Data analysis: ET, CH, FI, and OY. Providing reagents: JF, KF, NT, AR, and GA. Primary writing of the manuscript: ET and OY. Reviewing and revising the manuscript: ET, CH, FI, MH, JF, PK, KF, NT, AR, GA, and OY. All authors contributed to the article and approved the submitted version.

## Funding

Funding was provided by AIDS Healthcare Foundation and private philanthropic donors (including William Moses, Mari Edelman, Beth Friedman, Dana and Matt Walden, Kathleen Poncher, Scott Z. Burns, and Gwyneth Paltrow and Brad Falchuk), with additional infrastructure support from the UCLA AIDS Institute Center for AIDS Research (NIH grant AI028697), James B. Pendleton Trust, and McCarthy Foundation. Ancillary support was provided by Thermo Fisher (represented by Russ Pong), who provided access to the Attune Flow Cytometer and gifted fluorescent tagged antibodies. We appreciate the collaboration of Lisa Kelly and Irene Trovato with HyClone products from Cytiva (Logan, UT, www.Cytiva.com). The funders were not involved in the study design, collection, analysis, interpretation of data, the writing of this article or the decision to submit it for publication.

## Conflict of Interest

The authors declare that the research was conducted in the absence of any commercial or financial relationships that could be construed as a potential conflict of interest.

## Publisher’s Note

All claims expressed in this article are solely those of the authors and do not necessarily represent those of their affiliated organizations, or those of the publisher, the editors and the reviewers. Any product that may be evaluated in this article, or claim that may be made by its manufacturer, is not guaranteed or endorsed by the publisher.

## References

[1] Cromer D Juno JA Khoury D Reynaldi A Wheatley AK Kent SJ. Prospects for Durable Immune Control of SARS-CoV-2 and Prevention of Reinfection. Nat Rev Immunol (2021) 21:395–404. doi: 10.1038/s41577-021-00550-x 33927374 PMC8082486

[2] Sadarangani M Marchant A Kollmann TR. Immunological Mechanisms of Vaccine-Induced Protection Against COVID-19 in Humans. Nat Rev Immunol (2021) 21:475–84. doi: 10.1038/s41577-021-00578-z PMC8246128 34211186

[3] Dougan M Nirula A Azizad M Mocherla B Gottlieb RL Chen P. Bamlanivimab Plus Etesevimab in Mild or Moderate Covid-19. N Engl J Med (2021) 385:1382–92. doi: 10.1056/NEJMoa2102685 PMC8314785 34260849

[4] Libster R Perez Marc G Wappner D Coviello S Bianchi A Braem V. Early High-Titer Plasma Therapy to Prevent Severe Covid-19 in Older Adults. N Engl J Med (2021) 384:610–8. doi: 10.1056/NEJMoa2033700 PMC7793608 33406353

[5] O'brien MP Forleo-Neto E Musser BJ Isa F Chan KC Sarkar N. Subcutaneous REGEN-COV Antibody Combination for Covid-19 Prevention. medRxiv (2021). doi: 10.1101/2021.06.14.21258567

[6] Agarwal A Mukherjee A Kumar G Chatterjee P Bhatnagar T Malhotra P. Convalescent Plasma in the Management of Moderate Covid-19 in Adults in India: Open Label Phase II Multicentre Randomised Controlled Trial (PLACID Trial). BMJ (2020) 371:m3939. doi: 10.1136/bmj.m3939 33093056 PMC7578662

[7] Simonovich VA Burgos Pratx LD Scibona P Beruto MV Vallone MG Vázquez C. A Randomized Trial of Convalescent Plasma in Covid-19 Severe Pneumonia. New Engl J Med (2020) 384:619–29. doi: 10.1056/NEJMoa2031304 PMC7722692 33232588

[8] Janiaud P Axfors C Schmitt AM Gloy V Ebrahimi F Hepprich M. Association of Convalescent Plasma Treatment With Clinical Outcomes in Patients With COVID-19: A Systematic Review and Meta-Analysis. JAMA (2021) 325:1185–95. doi: 10.1001/jama.2021.2747 PMC7911095 33635310

[9] Stankov MV Cossmann A Bonifacius A Dopfer-Jablonka A Ramos GM Godecke N. Humoral and Cellular Immune Responses Against SARS-CoV-2 Variants and Human Coronaviruses After Single BNT162b2 Vaccination. Clin Infect Dis (2021) 73:2000–8. doi: 10.1101/2021.04.16.21255412 PMC8384414 34134134

[10] Chen G Wu D Guo W Cao Y Huang D Wang H. Clinical and Immunological Features of Severe and Moderate Coronavirus Disease 2019. J Clin Invest (2020) 130:2620–9. doi: 10.1172/JCI137244 PMC7190990 32217835

[11] Braun J Loyal L Frentsch M Wendisch D Georg P Kurth F. SARS-CoV-2-Reactive T Cells in Healthy Donors and Patients With COVID-19. Nature (2020) 587:270–4. doi: 10.1038/s41586-020-2598-9 32726801

[12] Grifoni A Weiskopf D Ramirez SI Mateus J Dan JM Moderbacher CR. Targets of T Cell Responses to SARS-CoV-2 Coronavirus in Humans With COVID-19 Disease and Unexposed Individuals. Cell (2020) 181:1489–501.e1415. doi: 10.1016/j.cell.2020.05.015 32473127 PMC7237901

[13] Weiskopf D Schmitz KS Raadsen MP Grifoni A Okba NMA Endeman H. Phenotype and Kinetics of SARS-CoV-2-Specific T Cells in COVID-19 Patients With Acute Respiratory Distress Syndrome. Sci Immunol (2020) 5:eabd2071. doi: 10.1126/sciimmunol.abd2071 32591408 PMC7319493

[14] Le Bert N Clapham HE Tan AT Chia WN Tham CYL Lim JM. Highly Functional Virus-Specific Cellular Immune Response in Asymptomatic SARS-CoV-2 Infection. J Exp Med (2021) 218:e20202617. doi: 10.1084/jem.20202617 33646265 PMC7927662

[15] Wyllie D Jones HE Mulchandani R Trickey A Taylor-Phillips S Brooks T. SARS-CoV-2 Responsive T Cell Numbers and Anti-Spike IgG Levels Are Both Associated With Protection From COVID-19: A Prospective Cohort Study in Keyworkers. medRxiv (2021) 2020.2011.2002.20222778. doi: 10.1101/2020.11.02.20222778

[16] Liao M Liu Y Yuan J Wen Y Xu G Zhao J. Single-Cell Landscape of Bronchoalveolar Immune Cells in Patients With COVID-19. Nat Med (2020) 26:842–4. doi: 10.1038/s41591-020-0901-9 32398875

[17] Mcmahan K Yu J Mercado NB Loos C Tostanoski LH Chandrashekar A. Correlates of Protection Against SARS-CoV-2 in Rhesus Macaques. Nature (2020) 590:630–4. doi: 10.1038/s41586-020-03041-6 PMC7906955 33276369

[18] Kared H Redd AD Bloch EM Bonny TS Sumatoh H Kairi F. SARS-CoV-2-Specific CD8+ T Cell Responses in Convalescent COVID-19 Individuals. J Clin Invest (2021) 131:e145476. doi: 10.1172/JCI145476 PMC7919723 33427749

[19] Tan AT Linster M Tan CW Le Bert N Chia WN Kunasegaran K. Early Induction of Functional SARS-CoV-2-Specific T Cells Associates With Rapid Viral Clearance and Mild Disease in COVID-19 Patients. Cell Rep (2021) 34:108728. doi: 10.1016/j.celrep.2021.108728 33516277 PMC7826084

[20] Polack FP Thomas SJ Kitchin N Absalon J Gurtman A Lockhart S. Safety and Efficacy of the BNT162b2 mRNA Covid-19 Vaccine. N Engl J Med (2020) 383:2603–15. doi: 10.1056/NEJMoa2034577 PMC7745181 33301246

[21] Baden LR El Sahly HM Essink B Kotloff K Frey S Novak R. Efficacy and Safety of the mRNA-1273 SARS-CoV-2 Vaccine. N Engl J Med (2021) 384:403–16. doi: 10.1056/NEJMoa2035389 PMC7787219 33378609

[22] Abu-Raddad LJ Chemaitelly H Butt AA. Effectiveness of the BNT162b2 Covid-19 Vaccine Against the B.1.1.7 and B.1.351 Variants. N Engl J Med (2021) 385:187–89. doi: 10.1056/NEJMc2104974 PMC8117967 33951357

[23] Greaney AJ Loes AN Crawford KHD Starr TN Malone KD Chu HY. Comprehensive Mapping of Mutations in the SARS-CoV-2 Receptor-Binding Domain That Affect Recognition by Polyclonal Human Plasma Antibodies. Cell Host Microbe (2021) 29:463–76.e466. doi: 10.1016/j.chom.2021.02.003 33592168 PMC7869748

[24] Kustin T Harel N Finkel U Perchik S Harari S Tahor M. Evidence for Increased Breakthrough Rates of SARS-CoV-2 Variants of Concern in BNT162b2-mRNA-Vaccinated Individuals. Nat Med (2021) 27:1379–84. doi: 10.1101/2021.04.06.21254882 PMC8363499 34127854

[25] Madhi SA Baillie V Cutland CL Voysey M Koen AL Fairlie L. Efficacy of the ChAdOx1 Ncov-19 Covid-19 Vaccine Against the B.1.351 Variant. N Engl J Med (2021) 384:1885–98. doi: 10.1056/NEJMoa2102214 PMC7993410 33725432

[26] Haas EJ Angulo FJ Mclaughlin JM Anis E Singer SR Khan F. Impact and Effectiveness of mRNA BNT162b2 Vaccine Against SARS-CoV-2 Infections and COVID-19 Cases, Hospitalisations, and Deaths Following a Nationwide Vaccination Campaign in Israel: An Observational Study Using National Surveillance Data. Lancet (2021) 397:1819–29. doi: 10.1016/S0140-6736(21)00947-8 PMC8099315 33964222

[27] Grifoni A Sidney J Zhang Y Scheuermann RH Peters B Sette A. A Sequence Homology and Bioinformatic Approach Can Predict Candidate Targets for Immune Responses to SARS-CoV-2. Cell Host Microbe (2020) 27:671–80.e672. doi: 10.1016/j.chom.2020.03.002 32183941 PMC7142693

[28] Ibarrondo FJ Wilson SB Hultin LE Shih R Hausner MA Hultin PM. Preferential Depletion of Gut CD4-Expressing iNKT Cells Contributes to Systemic Immune Activation in HIV-1 Infection. Mucosal Immunol (2013) 6:591–600. doi: 10.1038/mi.2012.101 23149661 PMC3865278

[29] Ibarrondo FJ Hofman C Fulcher JA Goodman-Meza D Mu W Hausner MA. Primary, Recall, and Decay Kinetics of SARS-CoV-2 Vaccine Antibody Responses. ACS Nano (2021) 7:11180–91. doi: 10.1021/acsnano.1c03972 34159781

[30] Ibarrondo FJ Anton PA Fuerst M Ng HL Wong JT Matud J. Parallel Human Immunodeficiency Virus Type 1-Specific CD8+ T-Lymphocyte Responses in Blood and Mucosa During Chronic Infection. J Virol (2005) 79:4289–97. doi: 10.1128/JVI.79.7.4289-4297.2005 PMC1061549 15767429

[31] Sabado RL Kilpatrick S Ali A Dagarag M Ng HL Cao H. Detection of HIV-1-Specific CTL Responses in Clade B Infection With Clade C Peptides and Not Clade B Consensus Peptides. J Immunol Methods (2005) 296:1–10. doi: 10.1016/j.jim.2004.09.017 15680145

[32] Yang OO Daar ES Jamieson BD Balamurugan A Smith DM Pitt JA. Human Immunodeficiency Virus Type 1 Clade B Superinfection: Evidence for Differential Immune Containment of Distinct Clade B Strains. J Virol (2005) 79:860–8. doi: 10.1128/JVI.79.2.860-868.2005 PMC538553 15613314

[33] Yang OO Daar ES Ng HL Shih R Jamieson BD. Increasing CTL Targeting of Conserved Sequences During Early HIV-1 Infection Is Correlated to Decreasing Viremia. AIDS Res Hum Retroviruses (2011) 27:391–8. doi: 10.1089/aid.2010.0183 PMC3101083 21087140

[34] Jones N Agrawal D Elrefaei M Hanson A Novitsky V Wong JT. Evaluation of Antigen-Specific Responses Using In Vitro Enriched T Cells. J Immunol Methods (2003) 274:139–47. doi: 10.1016/S0022-1759(02)00510-0 12609540

[35] Lineburg KE Srihari S Altaf M Swaminathan S Panikkar A Raju J. Rapid Detection of SARS-CoV-2-Specific Memory T-Cell Immunity in Recovered COVID-19 Cases. Clin Transl Immunol (2020) 9:e1219. doi: 10.1002/cti2.1219 PMC7720530 33312565

[36] Peng Y Mentzer AJ Liu G Yao X Yin Z Dong D. Broad and Strong Memory CD4(+) and CD8(+) T Cells Induced by SARS-CoV-2 in UK Convalescent Individuals Following COVID-19. Nat Immunol (2020) 21:1336–45. doi: 10.1038/s41590-020-0782-6 PMC7611020 32887977

[37] Bonifacius A Tischer-Zimmermann S Dragon AC Gussarow D Vogel A Krettek U. COVID-19 Immune Signatures Reveal Stable Antiviral T Cell Function Despite Declining Humoral Responses. Immunity (2021) 54:340–54.e346. doi: 10.1016/j.immuni.2021.01.008 33567252 PMC7871825

[38] Laing AG Lorenc A Del Molino Del Barrio I Das A Fish M Monin L. A Dynamic COVID-19 Immune Signature Includes Associations With Poor Prognosis. Nat Med (2020) 26:1623–35. doi: 10.1038/s41591-020-1038-6 32807934

[39] Sadeghi A Tahmasebi S Mahmood A Kuznetsova M Valizadeh H Taghizadieh A. Th17 and Treg Cells Function in SARS-CoV2 Patients Compared With Healthy Controls. J Cell Physiol (2021) 236:2829–39. doi: 10.1002/jcp.30047 32926425

[40] Le Gros G Erard F. Non-Cytotoxic, IL-4, IL-5, IL-10 Producing CD8+ T Cells: Their Activation and Effector Functions. Curr Opin Immunol (1994) 6:453–7. doi: 10.1016/0952-7915(94)90127-9 7917114

[41] Schwaiger S Wolf AM Robatscher P Jenewein B Grubeck-Loebenstein B. IL-4-Producing CD8+ T Cells With a CD62L++(bright) Phenotype Accumulate in a Subgroup of Older Adults and Are Associated With the Maintenance of Intact Humoral Immunity in Old Age. J Immunol (2003) 170:613–9. doi: 10.4049/jimmunol.170.1.613 12496450

[42] Machura E Mazur B Rusek-Zychma M Barć-Czarnecka M. Cytokine Production by Peripheral Blood CD4+ and CD8+ T Cells in Atopic Childhood Asthma. Clin Dev Immunol (2010) 2010:606139. doi: 10.1155/2010/606139 21197090 PMC3004408

[43] Baek HJ Zhang L Jarvis LB Gaston JSH. Increased IL-4+ CD8+ T Cells in Peripheral Blood and Autoreactive CD8+ T Cell Lines of Patients With Inflammatory Arthritis. Rheumatology (2008) 47:795–803. doi: 10.1093/rheumatology/ken089 18390584

[44] Le Bert N Tan AT Kunasegaran K Tham CYL Hafezi M Chia A. SARS-CoV-2-Specific T Cell Immunity in Cases of COVID-19 and SARS, and Uninfected Controls. Nature (2020) 584:457–62. doi: 10.1038/s41586-020-2550-z 32668444

[45] Ansari A Arya R Sachan S Jha SN Kalia A Lall A. Immune Memory in Mild COVID-19 Patients and Unexposed Donors Reveals Persistent T Cell Responses After SARS-CoV-2 Infection. Front Immunol (2021) 12:636768. doi: 10.3389/fimmu.2021.636768 33777028 PMC7991090

[46] Cassaniti I Percivalle E Bergami F Piralla A Comolli G Bruno R. SARS-CoV-2 Specific T-Cell Immunity in COVID-19 Convalescent Patients and Unexposed Controls Measured by Ex Vivo ELISpot Assay. Clin Microbiol Infect (2021) 27:1029–34. doi: 10.1016/j.cmi.2021.03.010 PMC8016542 33813122

[47] Dan JM Mateus J Kato Y Hastie KM Yu ED Faliti CE. Immunological Memory to SARS-CoV-2 Assessed for Up to 8 Months After Infection. Science (2021) 371:587. doi: 10.1126/science.abf4063 PMC7919858 33408181

[48] Ferretti AP Kula T Wang Y Nguyen DMV Weinheimer A Dunlap GS. Unbiased Screens Show CD8(+) T Cells of COVID-19 Patients Recognize Shared Epitopes in SARS-CoV-2 That Largely Reside Outside the Spike Protein. Immunity (2020) 53:1095–107.e1093. doi: 10.1016/j.immuni.2020.10.006 33128877 PMC7574860

[49] Thieme CJ Anft M Paniskaki K Blazquez-Navarro A Doevelaar A Seibert FS. Robust T Cell Response Toward Spike, Membrane, and Nucleocapsid SARS-CoV-2 Proteins Is Not Associated With Recovery in Critical COVID-19 Patients. Cell Rep Med (2020) 1:100092. doi: 10.1016/j.xcrm.2020.100092 32904468 PMC7456276

[50] Schulien I Kemming J Oberhardt V Wild K Seidel LM Killmer S. Characterization of Pre-Existing and Induced SARS-CoV-2-Specific CD8(+) T Cells. Nat Med (2021) 27:78–85. doi: 10.1038/s41591-020-01143-2 33184509

[51] Redd AD Nardin A Kared H Bloch EM Pekosz A Laeyendecker O. CD8+ T Cell Responses in COVID-19 Convalescent Individuals Target Conserved Epitopes From Multiple Prominent SARS-CoV-2 Circulating Variants. medRxiv (2021). doi: 10.1101/2021.02.11.21251585 PMC8083629 34322559

[52] Sekine T Perez-Potti A Rivera-Ballesteros O Stralin K Gorin JB Olsson A. Robust T Cell Immunity in Convalescent Individuals With Asymptomatic or Mild COVID-19. Cell (2020) 183:158–68.e114. doi: 10.1016/j.cell.2020.08.017 32979941 PMC7427556

[53] Brand I Gilberg L Bruger J Garí M Wieser A Eser TM. Broad T Cell Targeting of Structural Proteins After SARS-CoV-2 Infection: High Throughput Assessment of T Cell Reactivity Using an Automated Interferon Gamma Release Assay. Front Immunol (2021) 12. doi: 10.3389/fimmu.2021.688436 PMC8173205 34093595

[54] Zuo J Dowell AC Pearce H Verma K Long HM Begum J. Robust SARS-CoV-2-Specific T Cell Immunity Is Maintained at 6 Months Following Primary Infection. Nat Immunol (2021) 22:620–6. doi: 10.1038/s41590-021-00902-8 PMC7610739 33674800

[55] Breton G Mendoza P Hagglof T Oliveira TY Schaefer-Babajew D Gaebler C. Persistent Cellular Immunity to SARS-CoV-2 Infection. J Exp Med (2021) 218:e20202515. doi: 10.1084/jem.20202515 33533915 PMC7845919

[56] Peng Y Felce SL Dong D Penkava F Mentzer AJ Yao X. An Immunodominant NP105-113-B*07:02 Cytotoxic T Cell Response Controls Viral Replication and Is Associated With Less Severe COVID-19 Disease. Nat Immunol (2022) 23:50–61. doi: 10.1038/s41590-021-01084-z 34853448 PMC8709787

[57] Bilich T Nelde A Heitmann JS Maringer Y Roerden M Bauer J. T Cell and Antibody Kinetics Delineate SARS-CoV-2 Peptides Mediating Long-Term Immune Responses in COVID-19 Convalescent Individuals. Sci Transl Med (2021) 13:eabf7517. doi: 10.1126/scitranslmed.abf7517 33723016 PMC8128286

[58] Ma T Ryu H Mcgregor M Babcock B Neidleman J Xie G. Protracted Yet Coordinated Differentiation of Long-Lived SARS-CoV-2-Specific CD8+ T Cells During COVID-19 Convalescence. bioRxiv (2021). doi: 10.1101/2021.04.28.441880 PMC8763019 34389625

[59] Bacher P Rosati E Esser D Martini GR Saggau C Schiminsky E. Low-Avidity CD4(+) T Cell Responses to SARS-CoV-2 in Unexposed Individuals and Humans With Severe COVID-19. Immunity (2020) 53:1258–71.e1255. doi: 10.1016/j.immuni.2020.11.016 33296686 PMC7689350

[60] Casado JL Vizcarra P Velasco H Hammerle J Mcgee A Fernandez-Escribano M. Progressive and Parallel Decline of Humoral and T Cell Immunity in Convalescent Health Care Workers With Asymptomatic or Mild-Moderate SARS-CoV-2 Infection. J Infect Dis (2021) 224:241–5. doi: 10.1093/infdis/jiab242 PMC8136002 33961690

[61] Ibarrondo FJ Fulcher JA Goodman-Meza D Elliott J Hofmann C Hausner MA. Rapid Decay of Anti-SARS-CoV-2 Antibodies in Persons With Mild Covid-19. N Engl J Med (2020) 383:1085–7. doi: 10.1056/NEJMc2025179 PMC7397184 32706954

[62] Thomas SJ Moreira ED Kitchin N Absalon J Gurtman A Lockhart S. Six Month Safety and Efficacy of the BNT162b2 mRNA COVID-19 Vaccine. medRxiv (2021) 2021.2007.2028.21261159. doi: 10.1101/2021.07.28.21261159

[63] Agrati C Castilletti C Goletti D Meschi S Sacchi A Matusali G. Coordinate Induction of Humoral and Spike Specific T-Cell Response in a Cohort of Italian Health Care Workers Receiving BNT162b2 mRNA Vaccine. Microorganisms (2021) 9:1315. doi: 10.3390/microorganisms9061315 34208751 PMC8235087

[64] Long Q-X Tang X-J Shi Q-L Li Q Deng H-J Yuan J. Clinical and Immunological Assessment of Asymptomatic SARS-CoV-2 Infections. Nat Med (2020) 26:1200–4. doi: 10.1038/s41591-020-0965-6 32555424

[65] Qu J Wu C Li X Zhang G Jiang Z Li X. Profile of IgG and IgM Antibodies Against Severe Acute Respiratory Syndrome Coronavirus 2 (SARS-CoV-2). Clin Infect Dis (2020) 71:2255–8. doi: 10.1093/cid/ciaa489 PMC7197626 32337590

[66] Roltgen K Powell AE Wirz OF Stevens BA Hogan CA Najeeb J. Defining the Features and Duration of Antibody Responses to SARS-CoV-2 Infection Associated With Disease Severity and Outcome. Sci Immunol (2020) 5:eabe0240. doi: 10.1126/sciimmunol.abe0240 33288645 PMC7857392

[67] Jackson LA Anderson EJ Rouphael NG Roberts PC Makhene M Coler RN. An mRNA Vaccine Against SARS-CoV-2 — Preliminary Report. N Engl J Med (2020) 383:1920–31. doi: 10.1056/NEJMoa2022483 PMC7377258 32663912

[68] Sahin U Muik A Derhovanessian E Vogler I Kranz LM Vormehr M. COVID-19 Vaccine BNT162b1 Elicits Human Antibody and TH1 T Cell Responses. Nature (2020) 586:594–9. doi: 10.1038/s41586-020-2814-7 32998157

[69] Angyal A Longet S Moore SC Payne RP Harding A Tipton T. T-Cell and Antibody Responses to First BNT162b2 Vaccine Dose in Previously Infected and SARS-CoV-2-Naive UK Health-Care Workers: A Multicentre Prospective Cohort Study. Lancet Microbe (2022) 3:e21–31. doi: 10.1016/S2666-5247(21)00275-5 PMC8577846 34778853

