# Supplementary material for: Dominant CD8+ T Cell Nucleocapsid Targeting in SARS-CoV-2 Infection and Broad Spike Targeting From Vaccination
Source: Front Immunol. 2022 Feb 22;13:835830. doi: 10.3389/fimmu.2022.835830 (PMC8902813; doi:10.3389/fimmu.2022.835830)
Supplement: Supplementary file 1 [file DataSheet_1.pdf]

Dominant CD8$^+$ T Cell Nucleocapsid Targeting in SARS-CoV-2 Infection and Broad Spike Targeting from Vaccination

(Supplemental Materials)

Ellie Taus[1], Christian Hofmann[2], F. Javier Ibarrondo[2], Mary Ann Hausner[2], Jennifer A. Fulcher[2], Paul Krogstad[1,3], Kathie G. Ferbas[2], Nicole H. Tobin[3], Anne W. Rimoin[4], Grace M. Aldrovandi[3], Otto O. Yang[2,5]*

[1] Department of Molecular and Medical Pharmacology, David Geffen School of Medicine, University of California, Los Angeles, Los Angeles, CA, USA
[2] Department of Medicine, David Geffen School of Medicine, University of California Los Angeles, Los Angeles, CA, USA
[3] Department of Pediatrics, David Geffen School of Medicine, University of California Los Angeles, Los Angeles, CA, USA
[4] Fielding School of Public Health, University of California Los Angeles, Los Angeles, CA, USA
[5] Department of Microbiology, Immunology, and Molecular Genetics, David Geffen School of Medicine, University of California Los Angeles, Los Angeles, CA, USA

*Corresponding author:

Otto Yang
BSRB 173, 615 Charles E Young Drive South, Los Angeles, CA 90095
Phone: 310-794-9491
Facsimile: 310-983-1067
Email: oyang@mednet.ucla.edu

SUPPLEMENTAL FIGURES

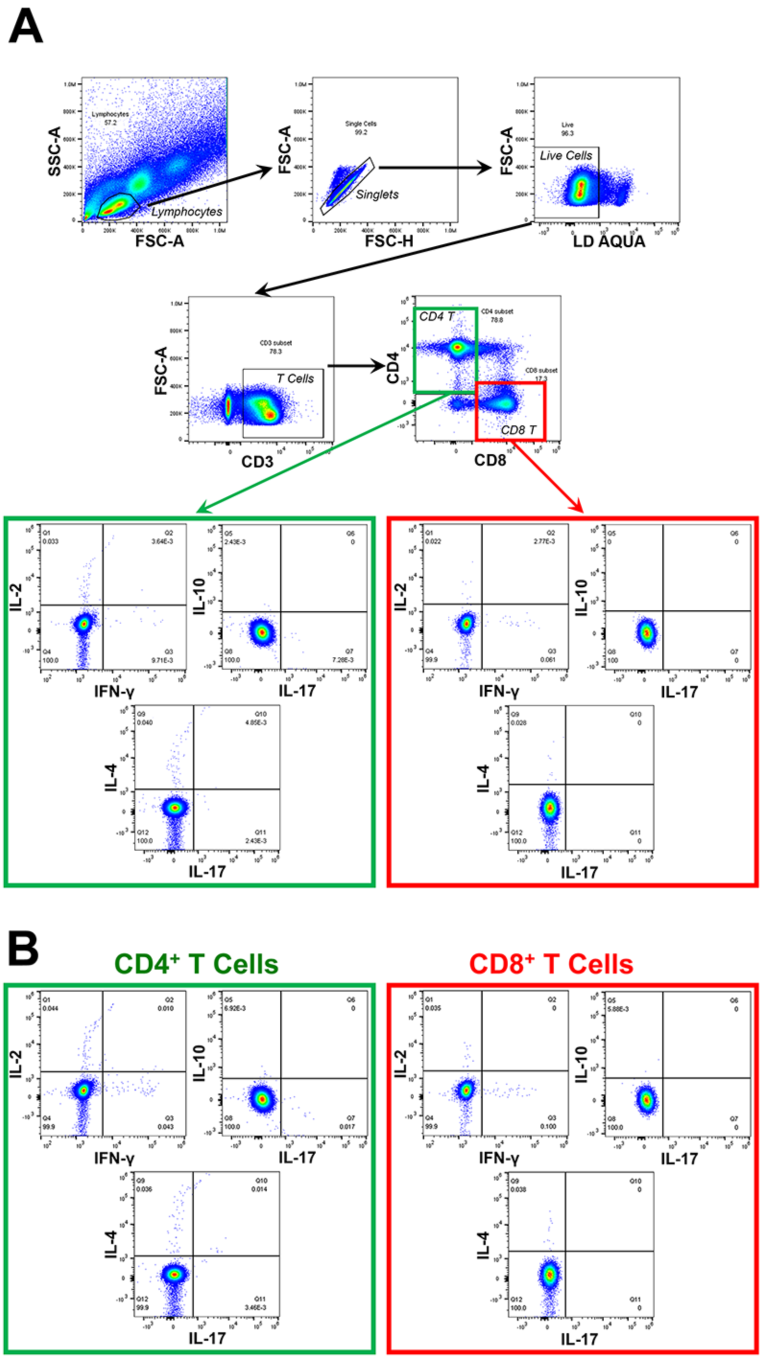

Supplemental Figure S1. Flow cytometry gating strategy and sample data. A. T cells were defined as $CD3^+$ cells within the live cell singlet lymphocyte population, and evaluated as $CD4^+$ and $CD8^+$ subsets. Unstimulated cell results are shown in the colored boxes reflecting the final gated cells. B. Representative megapool-stimulated cell results are shown.

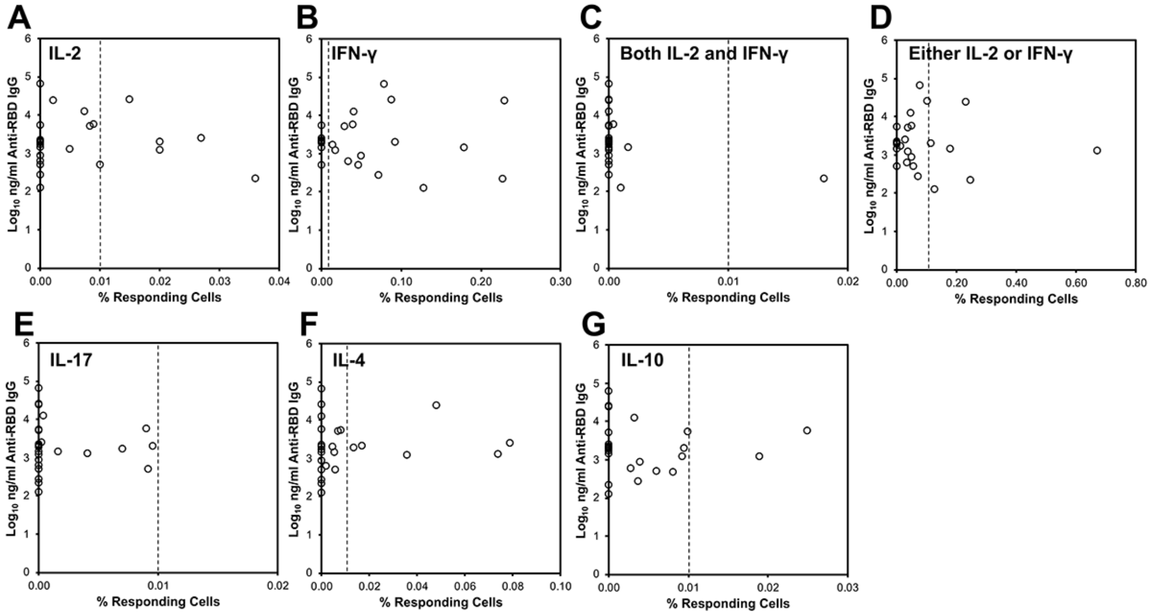

Supplemental Figure S2. Comparison of CD8$^+$ T cell cytokine responses against SARS-CoV-2 to serum anti-RBD antibody levels demonstrates no correlation. SARS-CoV-2-specific responses defined as in Figure 1 (x-axis) were compared to serum anti-RBD IgG antibody levels (y-axis). The vertical dotted line indicates 0.01% responding cells producing the indicated cytokine(s).

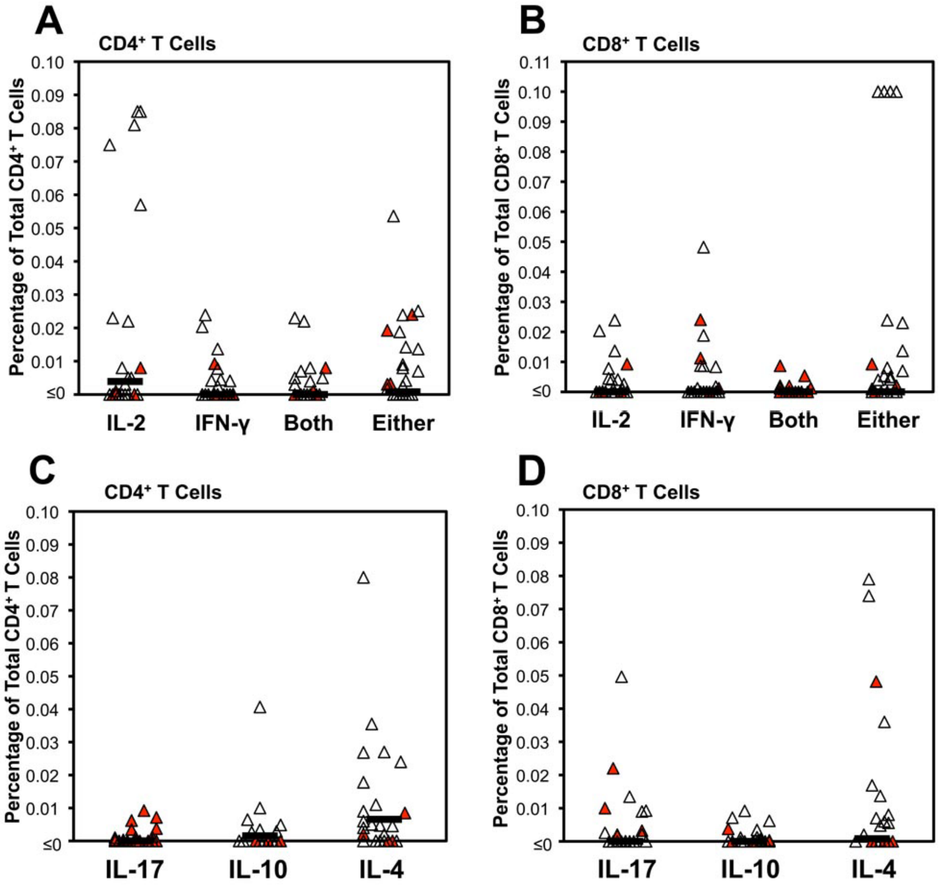

Supplemental Figure S3. Intracellular cytokine staining for T cell responses against SARS-CoV-2 early after infection (using pooled predicted CD8$^+$ T cell epitopes) for IL-2, IFN-γ, L-17, IL-10, and IL-4 yields similar results to those in Figure 1. Cytokine responses were defined as in Figure 1, except using pooled predicted CD8$^+$ T cell epitope peptides. Filled symbols indicate persons with severe infection.

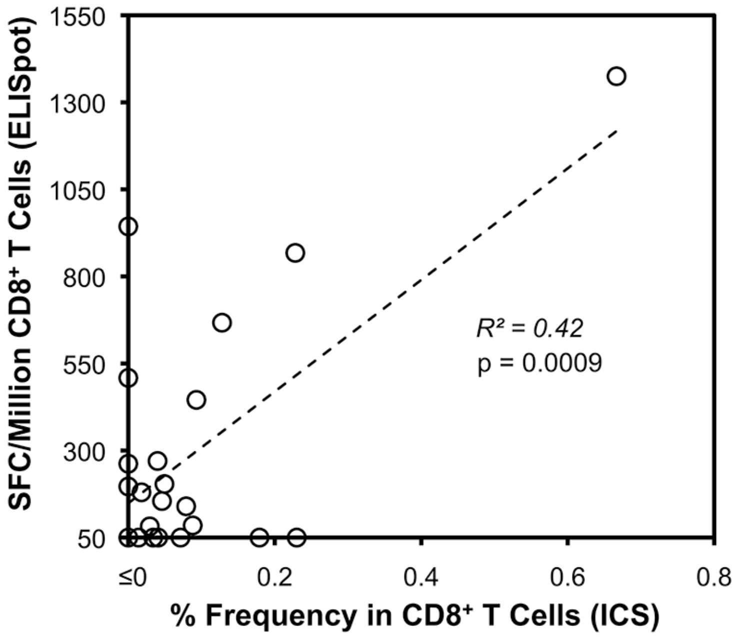

Supplemental Figure S4. Intracellular cytokine staining and ELISpot $CD8^+$ T cell responses correlate. For 24 persons in whom both intracellular IFN-γ staining (Figure 1) and IFN-γ ELISpot using spike-spanning peptides were performed (Figure 3), the results are plotted against each other. The Pearson correlation p value is indicated.

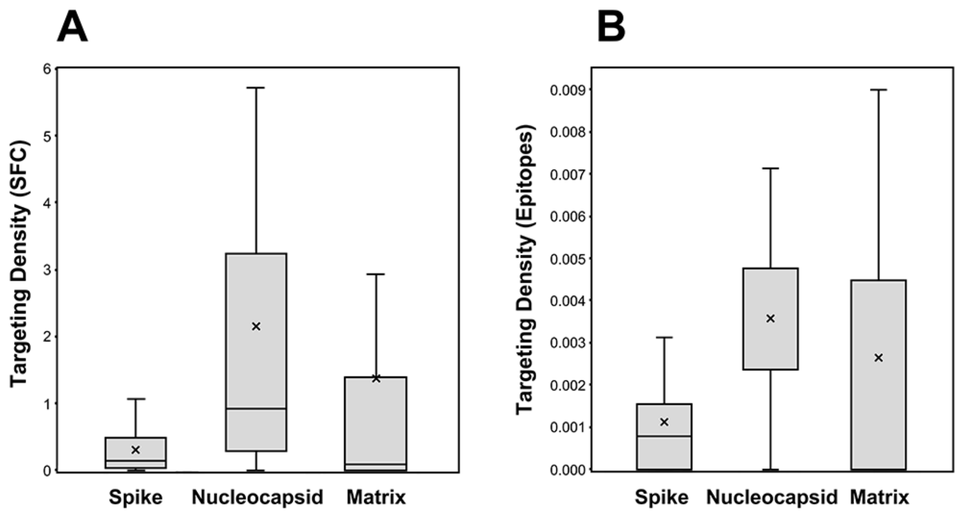

Supplemental Figure S5. The targeting density of SARS-CoV-2 CD8$^+$ T cell responses is highest against nucleocapsid. For the 44 persons in whom ELISpot responses were defined against spike, nucleocapsid, and matrix proteins (Figure 3), the densities of targeting in the context of protein size (1273 amino acids for spike, 419 amino acids for nucleocapsid, 222 amino acids for matrix) were calculated.

Panel A: Targeting density was defined as the ratio of SFC/10^6 CD8$^+$ T cells/# amino acids.

Panel B: Targeting density was defined as the ratio of epitopes (approximated by recognized peptide pools)/# amino acids.

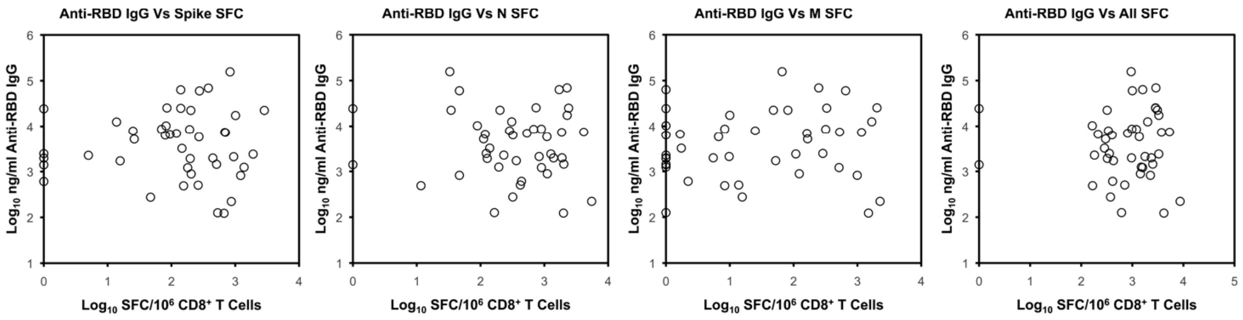

Supplemental Figure S6. CD8+ T cell responses by IFN-γ ELISpot against SARS-CoV-2 do not correlate to serum anti-RBD antibody levels. CD8+ T cell responses against spike, nucleocapsid (N), and matrix (M) depicted in Figure 3 were plotted against anti-RBD IgG levels.

SUPPLEMENTAL TABLES

**Initial Surface/Viability Stain Panel**		
**Target**	**Label**	**Catalog#, Company**
CD3	Super Bright 436	#62-0037-42, eBioscience, San Diego, CA
CD8	Super Bright 600	#63-0088-42, eBioscience, San Diego, CA
CD4	PE-Cy7	#25-0049-42, eBioscience, San Diego, CA
(Viability)	Fixable Aqua	#L34957, Invitrogen, Waltham, MA
**Intracellular Cytokine Stain Panel**		
**Target**	**Label**	**Catalog#, Company**
IL-4	PE	#130-091-647, Miltenyi Biotec, Bergisch Gladbach, Germany
IL-2	PerCP-Cy5.5	#506504, BioLegend, San Diego, CA
IL-10	APC	# 506807, BioLegend, San Diego, CA
IL-17	APC-eFluor	# 47-7179-42, eBioscience, San Diego, CA
IFN-ɣ	FITC	#506504, BioLegend, San Diego, CA

Supplemental Table S1. Antibodies utilized for surface and intracellular cytokine staining flow cytometry.

			Peptide Number		Amino Acid Number	
	Pool	# Peptides	First	Last	First	Last
**Spike Pools**	S1	15	1	15	1	115
	S2	15	16	30	106	220
	S3	15	31	45	211	325
	S4	15	46	60	316	430
	S5	15	61	75	421	535
	S6	15	76	90	526	640
	S7	15	91	105	631	745
	S8	15	106	120	736	850
	S9	15	121	135	841	955
	S10	15	136	150	946	1060
	S11	15	151	165	1051	1165
	S12	16	166	181	1156	1273
	Note: RBD is amino acids 319-541					
**Nucleocapsid Pools**	N1	15	1	15	1	115
	N2	15	16	30	106	220
	N3	15	31	45	211	325
	N4	14	46	59	316	419
**Matrix Pools**	M1	15	1	15	1	115
	M2	16	16	31	106	199
**Envelope Pool**	E	10	1	10	1	75

Supplemental Table S2. Peptide pools utilized for ELISpot mapping.
